# Effects of decongestion on nasal cavity air conditioning efficiency: a CFD cohort study

**DOI:** 10.1038/s41598-024-58758-5

**Published:** 2024-04-11

**Authors:** Qiwei Xiao, Alister J. Bates, Denis J. Doorly

**Affiliations:** 1 Center for Pulmonary Imaging Research, Cincinnati Children’s Hospital Medical Center, Cincinnati, OH USA; 2 Division of Pulmonary Medicine, Cincinnati Children’s Hospital Medical Center, Cincinnati, OH USA; 3 Department of Pediatrics, University of Cincinnati, Cincinnati, OH USA; 4 Department of Aeronautics, Imperial College London, South Kensington Campus, London, UK SW7 2AZ

**Keywords:** Translational research, Computational biophysics, Engineering, Biomedical engineering

## Abstract

Decongestion reduces blood flow in the nasal turbinates, enlarging the airway lumen. Although the enlarged airspace reduces the trans-nasal inspiratory pressure drop, symptoms of nasal obstruction may relate to nasal cavity air-conditioning. Thus, it is necessary to quantify the efficiency of nasal cavity conditioning of the inhaled air. This study quantifies both overall and regional nasal air-conditioning in a cohort of 10 healthy subjects using computational fluid dynamics simulations before and after nasal decongestion. The 3D virtual geometry model was segmented from magnetic resonance images (MRI). Each subject was under two MRI acquisitions before and after the decongestion condition. The effects of decongestion on nasal cavity air conditioning efficiency were modelled at two inspiratory flowrates: 15 and 30 L min^−1^ to represent restful and light exercise conditions. Results show inhaled air was both heated and humidified up to 90% of alveolar conditions at the posterior septum. The air-conditioning efficiency of the nasal cavity remained nearly constant between nostril and posterior septum but dropped significantly after posterior septum. In summary, nasal cavity decongestion not only reduces inhaled air added heat by 23% and added moisture content by 19%, but also reduces the air-conditioning efficiency by 35% on average.

## Introduction

Nasal cavity air-conditioning protects our delicate lung tissues from harm by heating and humidifying inhaled air close to alveolar conditions. The inner surface of the nasal cavity is covered with mucosa and these tissues serve as the heat and water source, typically supplying approximately 360 kcal of heat and 250–400 ml of water per day during the air-conditioning process^1,2^. A recent study has shown that heat and water transfer at the nasal surface is responsible for the perception of nasal airflow and when air conditioning is limited due to pathology, patients report the sensation of nasal obstruction, regardless of airflow^3^.

Investigations into nasal cavity air-conditioning have been conducted through both in-vivo and computational methods. Notably, Keck et al. mapped the spatial distribution of air conditioning within the nose using in-vivo measurements over a cohort of 50 healthy volunteers and found the inhaled air temperature significantly increased within the anterior nasal cavities and the humidification process was almost synchronous with the heating process^4,5^. Computational Fluid Dynamics (CFD) simulations revealed that even in extreme external temperatures, the nose is capable of heating inhaled air to near body temperature^6^. However, earlier CFD studies of nasal cavity air conditioning have used simplified models; others such as Lindemann et al. and Pless et al., did not incorporate water vapor transport^8,9^. Garcia et al. investigated the nasal cavity heat and water vapor transport between control and pathological subjects by assuming both constant cavity surface temperature and relative humidity^7^. A two-film theory was applied in simulating the heating of nasal cavity air by Kumahata et al. and Hanida et al. that evaluated the effects of latent heat, while the water transfer model assumed fully saturated conditions at the wall of the nasal cavity^8^. More recently, a study by Inthavong et al. applied a two-film theory to both the heat and water model in simulating the nasal cavity air-conditioning over a limited number of subjects^9^.

The nasal cycle, a natural physiological process, alternately decongests one side of the nasal cavity approximately every two hours, leading to anatomical variations that can affect air-conditioning efficiency^10^. Prior studies often utilized single geometric models per subject, based on the nasal anatomy at the time of imaging, although it has been shown considering the airway dynamics can make big difference in CFD predicted resutls^9,11–19^.

In this study, we aim to quantify the influence of anatomical variation on the efficiency of nasal air conditioning. By applying a nasal decongestant to both sides of the nasal cavity, we mimicked not only an extreme state of the nasal cycle using decongestant, representing the most decongested state possible, but also the use of decongestants is relevant for their practical application in temporarily alleviating nasal obstruction. This approach allowed us to measure the impact of nasal anatomy on air conditioning efficiency under both normal and decongested conditions in a paired sample study.

## Materials and methods

### Subjects

A cohort of 10 healthy volunteers aged between 21 and 38 were recruited. Ethical approval was granted by National Health Service Health Research Authority NRES Committee Southeast Coast-Surrey with reference number 06/Q0602/18. The image acquisition protocol and the translation of magnetic resonance images (MRI) to 3D virtual airway surfaces were the same as in previously published work^20^.

 Informed consent was obtained from each of the subjects included in this study. All methods were carried out in accordance with relevant guidelines and regulations.

### CFD simulations and boundary conditions

#### CFD simulations

The CFD software STAR-CCM+ (Siemens PLM Software, Plano, TX) was used to model the heat and water transported by the airflow through the nasal cavity. The flow computations were performed for inspiratory flowrates at 15 and 30 L min^−1^ using the methodology as described more fully in previous work. Briefly, the airflow through the nasal cavity was consider incompressible, so the mass conservation formula over a control volume can be written in the following form:

$$\nabla \cdot \left(\rho \overrightarrow{u}\right)=0$$

where $\nabla$ is divergence operator, $\overrightarrow{u}$ (m/s) means the velocity vector, $\rho\,({\text{kg}}\,{{\text{m}}}^{-3})$ is flow density. The momentum conservation can be expressed as follows:

$$\rho \left(\frac{\partial \overrightarrow{u}}{\partial t}+ \overrightarrow{u}\cdot \nabla \overrightarrow{u}\right)= -\nabla p+\mu {\nabla }^{2}\overrightarrow{u}$$

where $\mu$ (Pa s) is dynamic viscosity and ${\nabla }^{2}$ is Laplace operator. Both flowrates were simulated using a laminar model, because the flow characteristic in the majority of the nasal cavity is mainly laminar as shown by multiple previous studies^20–24^. The effects of gravity and buoyancy were neglected. The numerical schemes used for the temporal term is second order backward differentiation which involves the current and previous two time levels, and for the convection term the second-order upwind scheme was applied.

**Figure 1:**
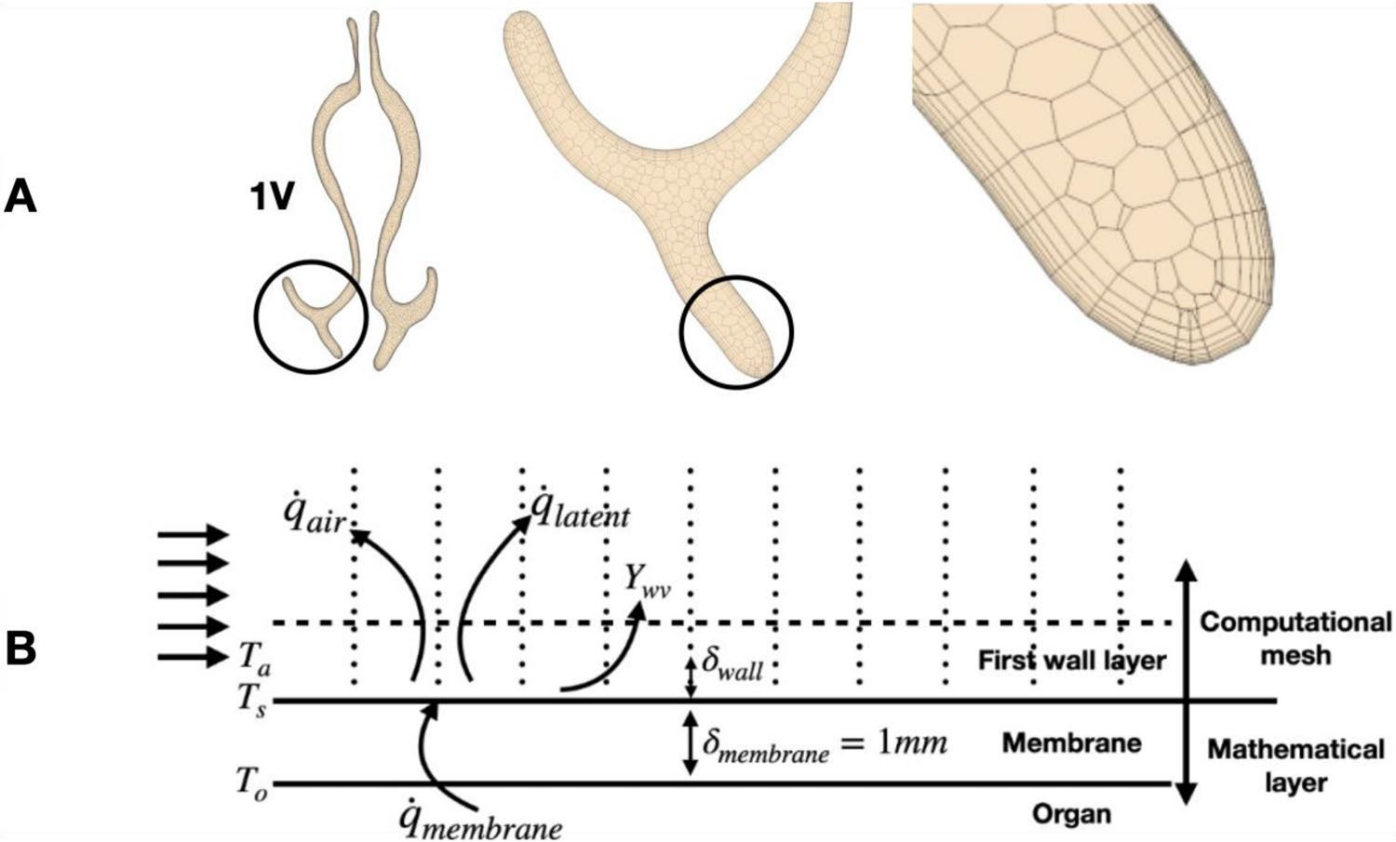
(**A**) A visual representation of polyhedral volume mesh created using STAR-CCM + on plane 1V (defined in Fig. 2) with the setting that gives 4 million volume meshes in total. (**B**) Diagram of the thermal and moisture conditions at the nasal cavity wall. ${{\text{T}}}_{{\text{O}}}$ represents the temperature at the surface between nasal tissue and mucosa, which is assumed at constant temperature of 34 °C. ${{\text{T}}}_{{\text{s}}}$ represents the temperature at the surface between the mucosa and the air. ${{\text{T}}}_{{\text{a}}}$ is the temperature of the air at the center of the first CFD volume mesh cell adjacent to the wall. Membrane represents a membrane layer which has the same properties as water.

**Figure 2:**
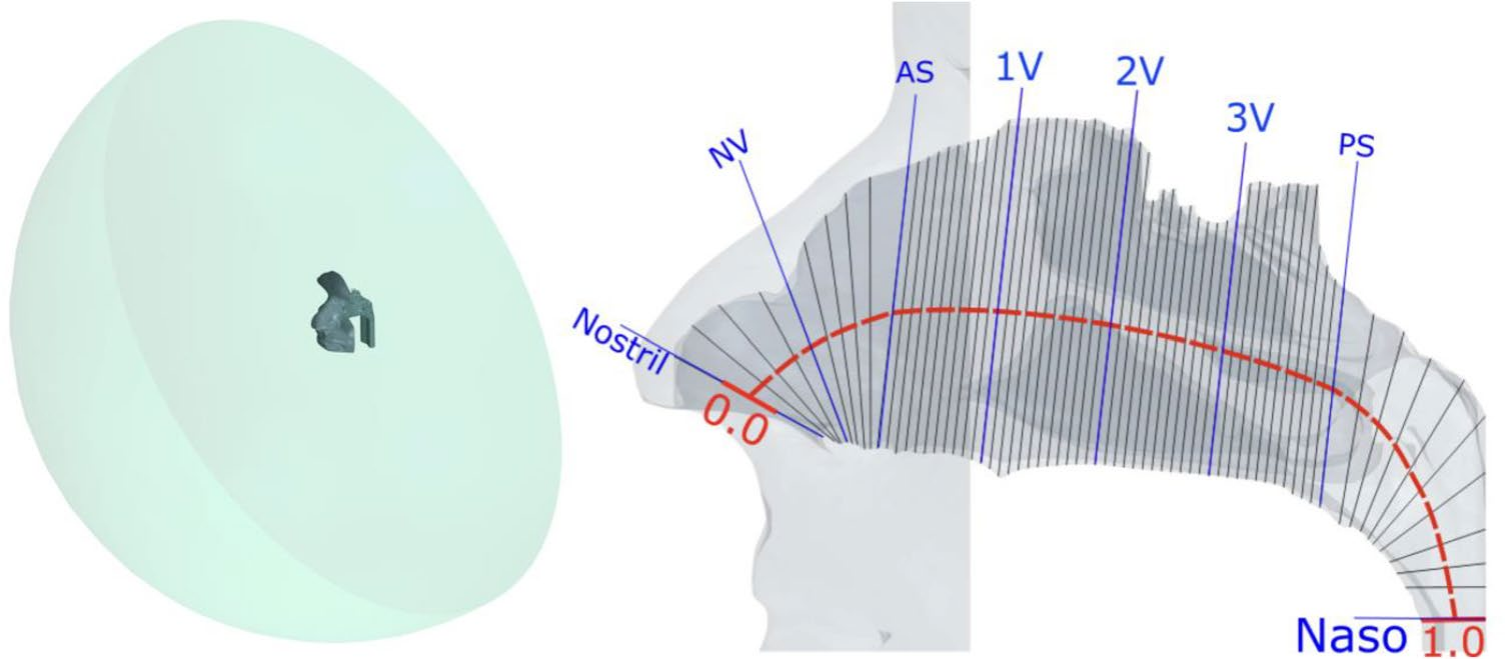
Left side shows the perspective view of a half meter sphere that was attached to the face, and extrusion at the end of nasopharynx with length of 50 times diameter. The right side shows the anatomy of a subject specific nasal cavity with cross-sectional planes as gray line overlay. There are several anatomical landmarks labelled by blue lines: Nostril, nasal valve (NV), anterior septum (AS), posterior septum (PS), and nasopharynx (Naso). 1V, 2V, 3V are three equal spaced landmarks between AS and PS. The overall centerline length was indicated by red dashed line. The normalized length of red dashed line scales between 0 and 1.

A mass flow inlet was specified at a far-removed external domain inlet and a pressure outlet condition (0 Pa gauge pressure) was specified at an extended nasopharyngeal outlet^20^. This setup of the boundary conditions is suitable because the pressure difference between locations, not the absolute pressure is the metric of interest in this study given the known inlet flowrate. There are 4 million total CFD polyhedral volume cells for each CFD simulation, and each simulation has 7 prism layers near the airway wall to account for both high flow and temperature gradient. The total thickness of prism layer is 0.3 mm, and the first layer is 0.01 mm with a stretch factor of 1.7 to allow a smooth transition of cells in between. Figure 1A demonstrates the mesh details at plane 1V (defined in Fig. 2) that was used for all the simulations in this study. More detailed mesh scenes can be found in the previous study on nasal airflow distribution in this cohort, as well as in the appendix section on mesh independence results^20^. Results using our model for heat and water transfer are compared with previous in-vivo measurements in the results section.

#### Heat and water vapor boundary conditions

The transport of heat and water vapor by the airflow may be described respectively by:

1 $$\frac{\partial T}{\partial t}+ \left(\overrightarrow{{\text{u}}}\cdot \nabla \right)T=\frac{k}{\uprho {C}_{p}}{\nabla }^{2}T$$

2 $$\frac{\partial {{\text{Y}}}_{\mathrm{\omega v}}}{\partial {\text{t}}}+\left(\overrightarrow{{\text{u}}}\cdot \nabla \right){Y}_{\mathrm{\omega v}}=D{\nabla }^{2}{Y}_{\mathrm{\omega v}}$$

where $T (K)$ is temperature, $\overrightarrow{u} ({\text{m}}/{\text{s}})$ is the flow velocity, $k ({\text{W}} {{\text{m}}}^{-1} {{\text{K}}}^{-1})$ thermal conductivity, $\rho ({\text{kg}}\, {{\text{m}}}^{-3})$ density, ${C}_{P} ({\text{J}} \,{{\text{kg}}}^{-1}\, {\text{K}})$ specific heat capacity, ${\nabla }^{2}$ is the Laplace operator, ${Y}_{\omega v}$ the water vapor fraction and $D ({{\text{cm}}}^{-2}\, {{\text{s}}}^{-1})$ the diffusion coefficient of water vapor in air. At the mucosal wall, a two-film model was employed to describe the exchange of heat and water with the mucosal surface.

Figure 1B shows schematically the modelling of heat and water vapor transfer from the nasal cavity wall into the inhaled air. The layer labeled “Organ” represents the nasal capillary bed, “Membrane” represents the layer of liquid mucus and the upper portion of the water supplying mucosal tissue on the nasal wall; the first wall layers represent the airway lumen as discretized in the CFD mesh (a detailed CFD mesh scene is shown in the Fig. 1A). The organ layer is responsible for providing the heat source in the model. This heat must then flow through the membrane to the air. The membrane layer is assumed to have the same specific heat capacity as water^25^. The temperature at the interface (${T}_{s}$) between the exposed surface of the nasal wall and the cavity air is determined by the resultant heat flux. The inhaled air temperature is represented by ${T}_{a}$, whilst ${T}_{o}$ refers to the temperature of the capillary bed, which is assumed at 34 °C based on previous studies^26–28^. The species boundary condition at the interface is assumed to be fully saturated at the local surface temperature.

The total amount of heat flux into the air ($\dot{q}_{air}$) is the sum of the flux from the membrane ($\dot{q}_{membrane}$) and the latent heat flux ($\dot{q}_{latent}$) carried by the water during evaporation (or condensation):

$$\dot{q}_{air}=\dot{q}_{latent}+\dot{q}_{membrane}$$

The fluxes $\dot{q}_{air}$, $\dot{q}_{latent}$ and $\dot{q}_{memb}$ are calculated using the equations below:

$$\dot{q}_{air}=-{k}_{air}\frac{{T}_{{\text{a}}}-{T}_{s}}{{\updelta }_{{\text{wall}}}}$$

$$\dot{q}_{latent}={L}_{{H}_{2}O}\cdot \dot{\upomega }$$

$$\dot{q}_{membrane}=-{k}_{membrane}\frac{{T}_{{\text{s}}}-{T}_{o}}{{\updelta }_{{\text{membrane}}}}$$

where $\dot{\upomega } ({\text{kg}} \,{{\text{m}}}^{-2}\, {{\text{s}}}^{-1})$ is the water mass flux between the fully saturated wall and the cavity air, and ${\updelta }_{wall} (m)$ is the half thickness of the first wall layer of the CFD mesh as labelled in Fig. 1B. The latent heat of water ${L}_{{{\text{H}}}_{2}{\text{O}}} ({\text{J}}\, {{\text{g}}}^{-1})$ is defined by the formula^29^:

$${L}_{{H}_{2}O}=2500.8-0.00006\cdot {T}_{s}^{3}+0.0016\cdot {T}_{s}^{2}-2.36\cdot {T}_{s},$$

where ${T}_{s}$ is in Celsius. Using Fick’s law of diffusion, $\dot{\upomega }$ is calculated as:

$$\dot{\omega }= -{\rho }_{fluid}\cdot D\cdot \frac{\partial {Y}_{wv}}{{\partial \delta }_{wall}}$$

where ${\rho }_{fluid}$ is the density of the mixture of air and water vapor. The mass fraction of water vapor under fully saturated conditions was quantified from an empirical fit of psychrometric data^30^ by the formula below.

$${Y}_{wv}=2\cdot 1{0}^{-5}{T}_{s}^{2}+0.0003{T}_{s}+0.0025$$

Heat flux and water flux into the air were determined by the surface temperature ${T}_{s}$, and were iteratively updated so that heat and water fluxes at the membrane and at the air were balanced at each time step. Combining the above equations, the surface temperate ${T}_{s}$ can be calculated as:

$${T}_{s}=\frac{{k}_{air}{T}_{a}{\delta}_{membrane}+{k}_{membrane}{T}_{o}{\delta}_{wall}-{L}_{H2O}\dot{\upomega }{\updelta }_{wall}{\updelta }_{membrane}}{{k}_{membrane}{\updelta }_{wall}+{k}_{air}{\updelta }_{membrane}}$$

For this cohort study, computations were performed with ${T}_{a}$ set to 25 °C, 50% relative humidity (corresponding to a mass fraction of 0.01125 and ${T}_{o}$ = 34 °C). The rendering of both the applied temperature and moisture boundary conditions can be found in appendix.

#### Geometric definition

The segmented 3D surface from the MRI scan of each subject includes anatomy from the face to the end of the nasopharynx. In addition, for each subject, an external half sphere (diameter = 0.5m) was attached to the face to ensure natural inflow profile and an extrusion (order of 50 diameter) was added from the end of nasopharynx to prevent reverse flow. The velocity of air approaching the nose during inhalation reduces rapidly, and an extension of this size is more than sufficient to ensure a natural flow profile develops at the entrance to the nose^21^. Although we have not investigated the minimum diameter needed, a smaller extension of order of 50mm diameter appears more than sufficient for modelling inhalation^21^. However, the extended domain facilities other expiratory flow studies and requires few additional elements. Extruding the outlet allows flow to stabilize and reduces the impact of artificial outlet boundary condition on the flow field. Figure 2 illustrates the reconstructed 3D surface of one subject with the anatomical landmarks used, as defined in our previous flow only study^20^.

### Measurements

#### Air temperature

The mean air temperature was calculated over each cross-sectional plane (Fig. 2 right) from anterior to posterior cavity in each subject; in addition, the cohort mean air temperature at each corresponding plane was also calculated.

#### Relative humidity and moisture content

Relative humidity (RH) is the ratio of the mass fraction of water vapor mixture to the mass fraction of water vapor if fully saturated, at the corresponding temperature and pressure. Lindemann et al., reported that RH can be 90% at 32 °C in the nasopharynx, however, RH is a temperature and pressure dependent metric, and it is not ideal for inter-subject comparisons, given the absolute amount of water vapor is the metric of interest. Therefore, moisture concentration was compared to alveolar conditions (100% RH at 37 °C), rather than local conditions, using a new quantity defined as the ratio between mass fraction of H_2_O at local conditions and the mass fraction of H_2_O at alveolar conditions, which was named moisture content (MC). The equation below shows the calculation of MC where ${{\text{Y}}}_{t}$ is the local mass fraction of water vapor, while ${{\text{Y}}}_{37\,^\circ{\rm C} }$ refers to the mass fraction of water vapor at alveolar conditions.

$$MC=100\cdot \frac{{{\text{Y}}}_{t}}{{{\text{Y}}}_{37^\circ{\rm C} }}$$

Thus, MC is suitable to quantify how much the water vapor is in the air compared to alveolar conditions, irrespective of local conditions.

Intra and inter-subject variations in MC were plotted in a similar manner to the temperature variations described above.

#### Film coefficients

To quantify the efficiency of heat and water transfer between the nasal cavity wall and inhaled air, heat and mass film coefficients were introduced. The equation below shows the calculation of the heat film coefficient. The left side of the equation $Q ({\text{W}})$ represents the heat flux while the right side of the equation is the product of heat film coefficient $h ({\text{W}}\,{{\text{m}}}^{2}\,{\text{K}})$, nasal cavity surface area $A\, ({{\text{m}}}^{2})$ and temperature gradient at the wall ($\Delta T$):

$$Q=h\cdot A\cdot\Delta T$$

Analogously to heat transfer, the mass film coefficient was calculated using the formula below, indicating water vapor flux is a product of mass film coefficient ${h}_{c} ({\text{m}} {{\text{s}}}^{-1})$, nasal cavity surface area $A ({{\text{m}}}^{2})$ and gradient of mass fraction of water vapor at the wall ($\Delta {c}_{A}$):

$${\Omega }_{A}{=h}_{c}A\Delta {c}_{A}$$

### Data analysis

The Wilcoxon signed-rank test was performed to evaluate the statistical significance of decongestion-associated changes in nasal cavity air-conditioning efficiency^31^. Following convention, a 95% confidence level was chosen to distinguish significant from non-significant results^32^. Box and whisker plots were used to illustrate the results.

## Results

### Comparison with in-vivo measurements

Keck et al. measured the temperature and humidity profile at multiple positions within nasal cavities of 50 subjects at 25 °C room temperature and 35% RH under restful breathing condition^4^. They reported the mean temperature and RH at the nasal valve, anterior turbinate and nasopharynx as shown in Table 1. To compare with the results of Keck et al., we conducted simulations under the same boundary temperature and humidity conditions and using 15 L min^−1^ as a representative restful breathing flow rate. Virtual probes were created at the anatomical similar locations in the CFD simulation. Given uncertainty in matching locations and the associated error ranges, results show reasonable agreement.

**Table 1:** The top 3 rows of the table show both mean temperature and relative humidity results over the cohort of 10 subjects at normal condition at three distinct anatomical locations in nasal cavity.

Exemplar subject	Nasal valve	Anterior turbinate	Nasopharynx
Temperature (°C)	27.9	28.9	32.4
Relative humidity (%)	68	83	98
Keck et al	Nasal valve	Anterior turbinate	nasopharynx
Temperature (°C)	28.9 ± 2.3	30.3 ± 1.6	32.6 ± 1.5
Relative humidity (%)	69.0 ± 6.5	78.7 ± 7.2	90.3 ± 5.3

The bottom 3 rows show the same information but from experimental measurements by Keck et al.^5^.

### Temperature and moisture content predictions

**Figure 3:**
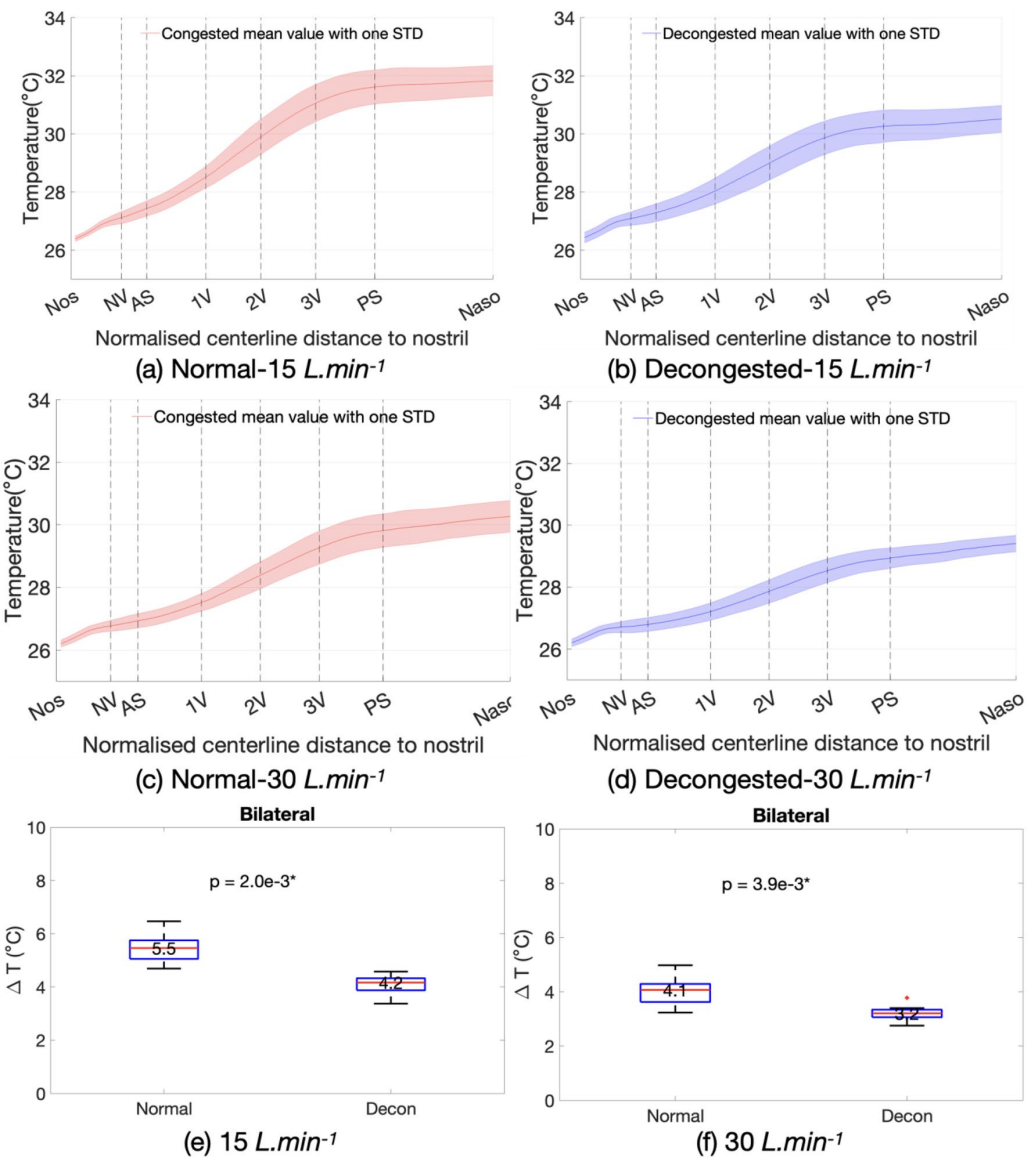
(**a**)–(**d**) The mean inhaled air temperature increase from nostril to nasopharynx over the cohort at both normal and decongested conditions with different flowrates as labelled. Panel e and f show the Wilcoxon-signed rank test result of temperature differences between nostril and nasopharynx at normal and decongested conditions at 15 and 30 L min^−1^ flowrates.

Figure 3a–d, shows results from CFD simulations of inhaled air temperature increase from anterior to posterior nasal cavity averaged over the cohort at both normal and decongested conditions. All simulations shared the same thermal and humidity inflow boundary conditions (25C, 50% RH). The solid line represents the mean value over the cohort and shaded area indicates the variations of one standard deviation.

At 15 L min^−1^ flowrate (Fig. 3a,b), decongestion reduced the amount of heat added to the inhaled air by 24% compared to normal condition on average for the cohort, with the final temperature at nasopharynx reduced from 31.8 to 30.5 °C After doubling the flowrate, decongestion reduced the same measurement of added heat by 22%, with temperature at nasopharynx reduced from 30.3 to 29.4 °C . Figure 3e,f showed the reduction in temperature to be statistically significant with a median reduction by 1.3 °C at flowrate of 15 L min^−1^, and 0.9 °C at flowrate of 30 L min^−1^ accordingly.

**Figure 4:**
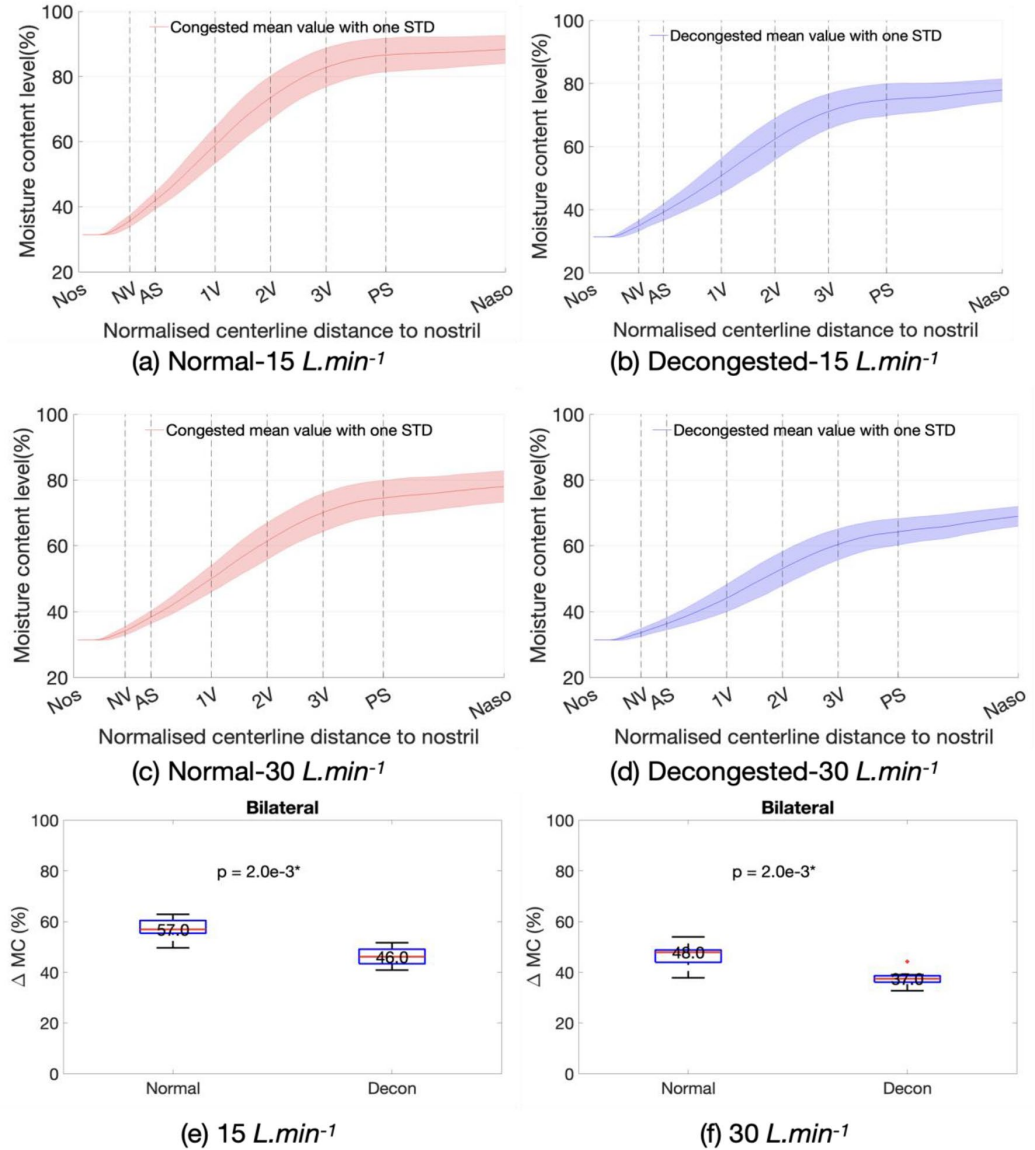
(**a**)–(**d**) The mean inhaled air moisture content increase from nostril to nasopharynx over the cohort at both normal and decongested conditions with different flowrates as labelled. Panel e and f show the Wilcoxon-signed rank test result of moisture content differences between nostril and nasopharynx at normal and decongested conditions at 15 and 30 L min^−1^ flowrates.

Similarly, in Fig. 4 from a–d the solid line indicates the mean value of the computed MC distribution in the nasal cavity over the cohort, with the shaded area representing variation of one standard deviation. Results show the MC added to the inhaled air was reduced by decongestion, respectively by 18% at 15 L min^−1^ flowrate and by 20% for 30 L min^−1^ flowrate at nasopharynx plane. Applying the Wilcoxon signed rank test confirmed the reductions in added MC to be statistically significant, with median reduction by 20% and 23% respectively. More detailed temperature and MC rendering in the nasal cavities are shown in the appendix.

### Heat and mass film coefficient

The heat film coefficient, *h,* is a measure of heating efficiency of the nose. Since the vast majority of heating occurs from the nostril to the posterior septum, the heat film coefficient was calculated over this region. Table 2 shows that on average across all subjects, the heat film coefficient is reduced by decongestant, as might be expected as a consequence of mean airflow speed reduction concomitant with increased cavity lumen area.

**Table 2:** Comparison of normal and decongested cohort-mean heat and mass film coefficients at two inspiratory flowrates.

Heat film coefficient *h* (W m^−2^ K^−1^), mean value of region between nostril and posterior septum			
Flowrate (L min^−1^)	Normal	Decongested	Change in *h* due to decongestion
15	125.4	78.0	–38%
30	174.0	112.1	–36%
Change in *h* due to increase in flowrate	40%	40%	

Mass film coefficient *h*_*c*_ (m s^−1^), mean value of region between nostril and posterior septum			
Flowrate (L min^−1^)	Normal	Decongested	Change in *h*_*c*_ due to decongestion
15	0.061	0.039	–36%
30	0.073	0.052	–29%
Change in *h*_*c*_ due to increase in flowrate	20%	33%	

Similarly, the mass film coefficient, *h*_*c*_, is a measure of the humidification efficiency of the nose. The mass film coefficient follows the same trends as the heat film coefficient in terms of changes with decongestion and flowrate. However, the changes in *h*_*c*_ are less than those for *h*, particularly those due to flowrate increases.

## Discussion

CFD was used to investigate the air conditioning capabilities of the nasal cavity in a cohort of 10 subjects, using in-vivo acquired data pre- and post-decongestion. The air-conditioning performance of the nasal cavity varies with the nasal cycle, and it is of both physiological and clinical interest to investigate the magnitude of the performance decrement caused by decongestion to further our understanding of nasal function.

Results indicated considerable variation in performance across the cohort, in both normal and decongested states. The distribution of modelled mucosal surface temperature (Fig. 3a–d) from anterior to posterior cavity show an initial rapid increase followed by a levelling off after the posterior septum as the surface area for heat exchange reduces. Similar trend is likewise found for the moisture content (MC). The computational modelling predicted a decongestion-associated reduction of added heat to inhaled air by 24% (15 L min^−1^) and 22% (30 L min^−1^) respectively. Similarly, the added MC to inhaled air were reduced by 18% (15 L min^−1^) and 20% (30 L min^−1^). All the decongestion-associated changes observed in this paired-sample study were found to be statistically significant.

The film coefficient is a parameter to quantify the efficiency of heat or mass transfer from nasal cavity inner wall to inhaled air. The results show decongestion can reduce the heat transfer efficiency by 37% and mass transfer efficiency by 33% for both lower and higher flowrates. In addition, doubling the flowrate can increase the heat (mass) transfer efficiency by 40% (25%) both before and after decongestion, which very likely due to relatively higher temperature gradient caused by fast moving flow near the cavity wall.

Limitations of the present study are firstly, due to the limitation of available computational power, only constant flowrates were simulated in this study. Future study should use subject specific breathing profile to increase the fidelity of the simulations. In addition, the simplified heat and water boundary conditions also can introduce errors especially by assuming fully saturated air at the nasal cavity wall and flow interface. Further work should focus on improving the boundary conditions as well as considering a far larger cohort than the present 10 subjects. It would also be of interest to extend the methodology reported here to consider pathological nasal anatomies. Lastly, the air conditioning performance of the nose depends on the external environment. An indication of the scale of this dependence is shown in the appendix for increasingly demanding external regimes and at an elevated flow rate of 30 L min^−1^. In the present work, we have studied 10 pairs of different nasal geometries at two flowrates to determine ranges of variation in air conditioning capability under the same external conditions. Extending such cohort studies to cover such broad range of conditions as in the appendix is a goal of further research, but brings with it a much enlarged burden of associated computational and data reduction requirements.

## Conclusion

In summary, the majority of the air conditioning (90%) process was completed before nasal posterior septum. The effect of decongestion can significantly reduce the final state of conditioned air at the level of the nasopharynx. On average, doubling the flowrate in the normal nose also lowers the air conditioning capability to similar levels as observed with decongestion. Specifically, the air conditioning efficiency of the nasal cavity can be reduced by 35% on average (including heating and humidifying) after decongestion, whilst doubling the flowrate can reduce the efficiency by 33% on average. This study has quantified how the changes in the nasal anatomy up to the nasal septum, which can be caused by decongestion, affects the conditioning of inhaled air.

## Supplementary Information

Supplementary Information.

## Data Availability

The datasets generated during and/or analyzed during the current study are available from the corresponding author on reasonable request.
