# Supplementary material for: Effects of decongestion on nasal cavity air conditioning efficiency: a CFD cohort study
Source: Sci Rep. 2024 Apr 11;14:8482. doi: 10.1038/s41598-024-58758-5 (PMC11375134; doi:10.1038/s41598-024-58758-5)
Supplement: Supplementary file 1 — Supplementary Information. [file 41598_2024_58758_MOESM1_ESM.pdf]

Effects of decongestion on nasal cavity air conditioning efficiency: a CFD cohort study

# Qiwei Xiao^1,2^, Alister J. Bates^1,2,3^, and Denis J. Doorly^4,*^

^1^Center for Pulmonary Imaging Research, Cincinnati Children’s Hospital Medical Center, Cincinnati, Ohio, USA

^2^Division of Pulmonary Medicine, Cincinnati Children’s Hospital Medical Center, Cincinnati, Ohio, USA

^3^Department of Pediatrics, University of Cincinnati, Cincinnati, Ohio, USA

^4^Department of Aeronautics, Imperial College London, South Kensington Campus, London, SW7 1AZ, United Kingdom

^*^d.doorly@imperial.ac.uk

# Abstract

Decongestion reduces blood flow in the nasal turbinates, enlarging the airway lumen. Although the enlarged airspace reduces the trans-nasal inspiratory pressure drop, symptoms of nasal obstruction may relate to nasal cavity air-conditioning. Thus, it is necessity to quantify how efficient nasal cavity conditioning the inhaled air.

This study quantifies both overall and regional nasal air-conditioning in a cohort of 10 healthy subjects using computational fluid dynamics simulations before and after nasal decongestion. The 3D virtual geometry model was segmented from magnet resonance images (MRI). Each subject was under two MRI acquisitions before and after decongestion condition. The effects of decongestion on nasal cavity air conditioning efficiency were modelled at two inspiratory flowrates: 15 and 30 $L.min^{-1}$ to represent restful and light exercise conditions.

Results show inhaled air was both heated and humidified up to 90% of alveolar conditions at the posterior septum. The air-conditioning efficiency of the nasal cavity remained nearly constant between nostril and posterior septum but dropped significantly after posterior septum. In summary, decongestion not only reduce nasal cavity added heat by 23% and added moisture content by 19% to inhaled air, but also reduce the air-conditioning efficiency by 35% on average.

# Appendix

## A: Temperature and Moisture Boundary Conditions:


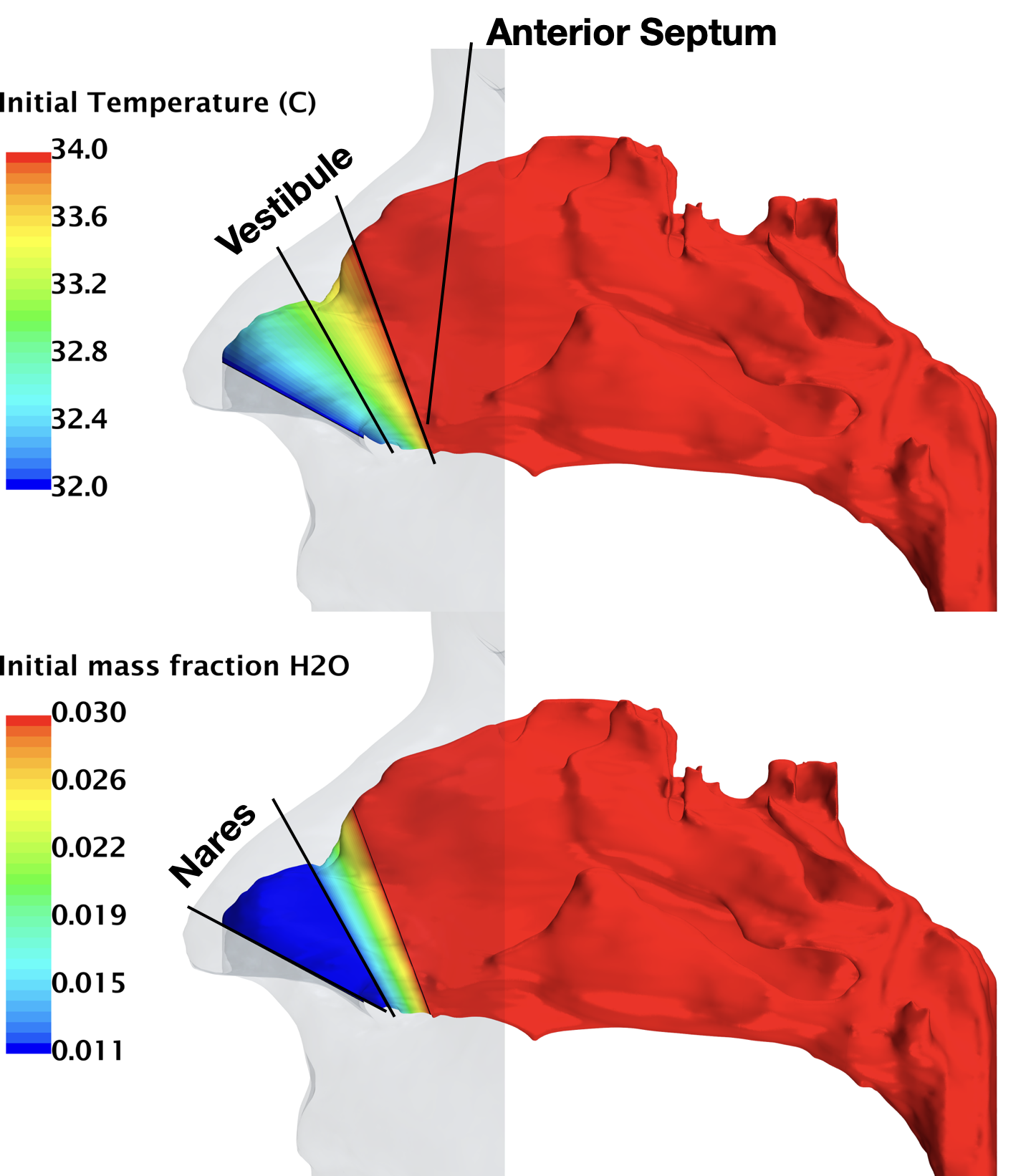


*Figure 1: The top and bottom figures show the boundary conditions for heat transfer and mass fraction of water respectively,*

The initial temperature gradient is established from the front of the nares to the end of the vestibule, with a starting temperature of 32 $^{\circ}C$ at the nares, incrementing linearly to 34 $^{\circ}C$ at the vestibule end, proportional to the distance covered.

For moisture, the boundary condition within the vestibule follows a similar linear transition, distinct from the nares, which maintains a constant moisture level equivalent to the incoming air’s moisture content.

As the simulation finished, with 25 $℃$ and 50% relative humidity inhaled air coming into the cavity, the inner surface of the nasal cavity settles with temperate and mass fraction of water as shown in the figures below:


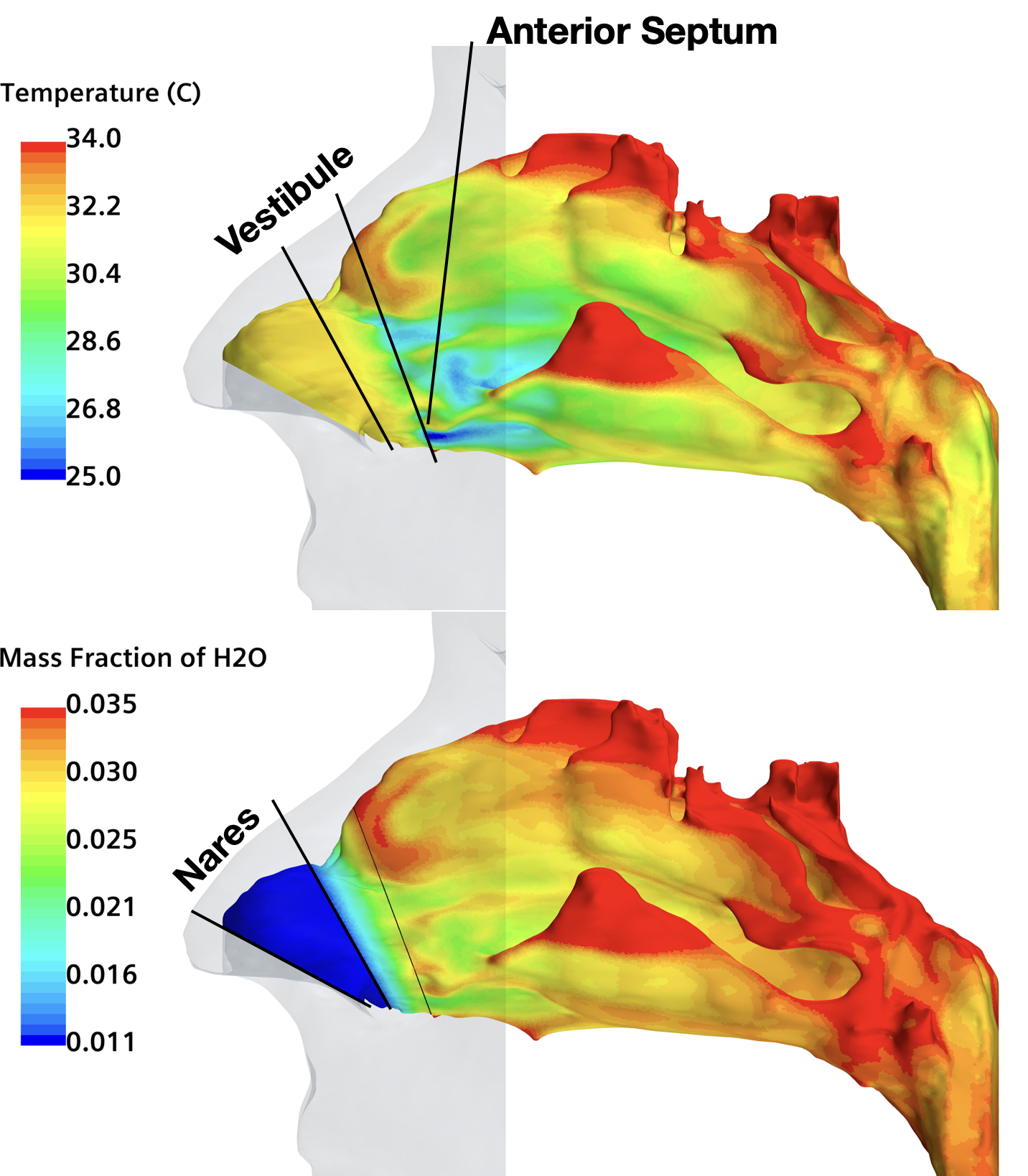


*Figure 2: The top and bottom figures show the simulation results of both temperature and mass fraction of water on the nasal cavity surface, when the subject inhales 25* $℃$ *and 50% relative humidity air at 30* $L/min$ *flowrate.*

## B: Mesh Independence

The mesh independence study uses as metrics (i) the total amount of water flux added between the anterior septum and posterior septum and (ii) the pressure loss as shown in the table below:

| Mesh amount (millions) | 1.9 | 2.6 | 3.0 | 4.0 | 5.2 | 6.5 |
| --- | --- | --- | --- | --- | --- | --- |
| Time step (ms) | 1 | 1 | 1 | 1 | 1 | 1 |
| Overall pressure loss (Pa) | 23.2 | 23.8 | 24.1 | 24.1 | 24.5 | 24.6 |
| Relative error (%) | 6 | 3 | 2 | 2 | 0.4 | 0 |
| Added water flux between AS and PS (kg/s) | 7.21e-6 | 7.34e-6 | 7.35e-6 | 7.37e-6 | 7.39e-6 | 7.4e-6 |
| Relative error (%) | 2.6 | 0.8 | 0.6 | 0.4 | 0.1 | 0 |

*Table 1: This table shows the added water flux between the anterior septum and posterior septum planes with the increase of total simulation mesh elements.*

The results in the table show the simulation with 4 million volume mesh elements and

1 ms timestep are sufficient for the metrics of interest considered in this study.

## C: Temperature and Moisture Content in the Nasal Cavity

To show how the temperature and moisture content levels vary from the anterior to posterior of the nasal cavity, we have created the following scenes at those landmark planes (defined in Figure 2 in the paper).


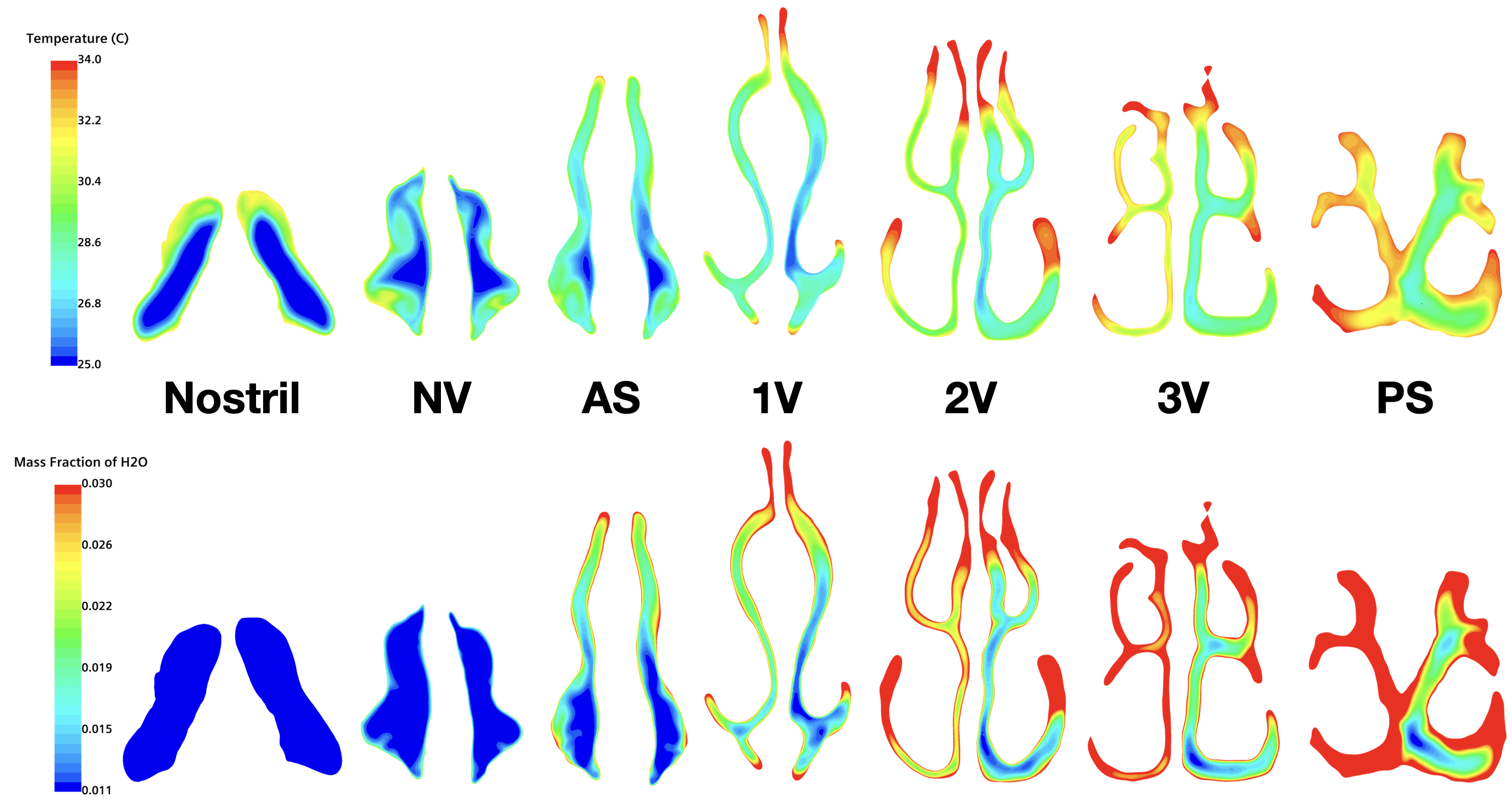


*Figure 3: Top row of the figure shows the temperature variations from the landmarks defined in Figure 2 of the manuscript. Nostril: plane at nostril, NV: plane at nasal valve, AS: plane at anterior septum, 1/2/3V vertical planes that evenly divide the nasal cavity of the region between AS and PS, PS: plane at posterior septum.*

Results from Figure 3 show both the temperature and mass fraction of water increase gradually from anterior to the posterior parts of the cavity. The narrow side of the nasal cavity shows slightly quicker increase of both temperature and mass fraction of water, which is due to the lower flow (higher resistance). This is a visual reflection of the results already presented in the line plots of Figure 3 and 4 in the manuscript.

## D: Effects of Ambient Conditions

To briefly investigate the effects of different external conditions on nasal cavity air-conditioning ability, we simulated 4 external conditions of one subject, whose cross-sectional area is a representative median nose over the cohort, at 30L/min flow rate. The table below shows the 4 different external conditions:

| Conditions | Temperature ($^{\circ}C$) | Relative humidity (%) | Absolute humidity (g.m^-3^) |
| --- | --- | --- | --- |
| 1 | 25 | 50 | 11.5 |
| 2 | 25 | 35 | 8.1 |
| 3 | 15 | 50 | 6.4 |
| 4 | 5 | 10 | 0.68 |

*Table 2: This table shows 4 different external conditions modeled for one specific subject. The absolute humidity indicates the real amount of water vapor in the air, rather than the relative metric relative humidity.*

Firstly, we plotted the cross-sectional area averaged temperature variation from nostril to posterior septum at those 4 different ambient conditions.


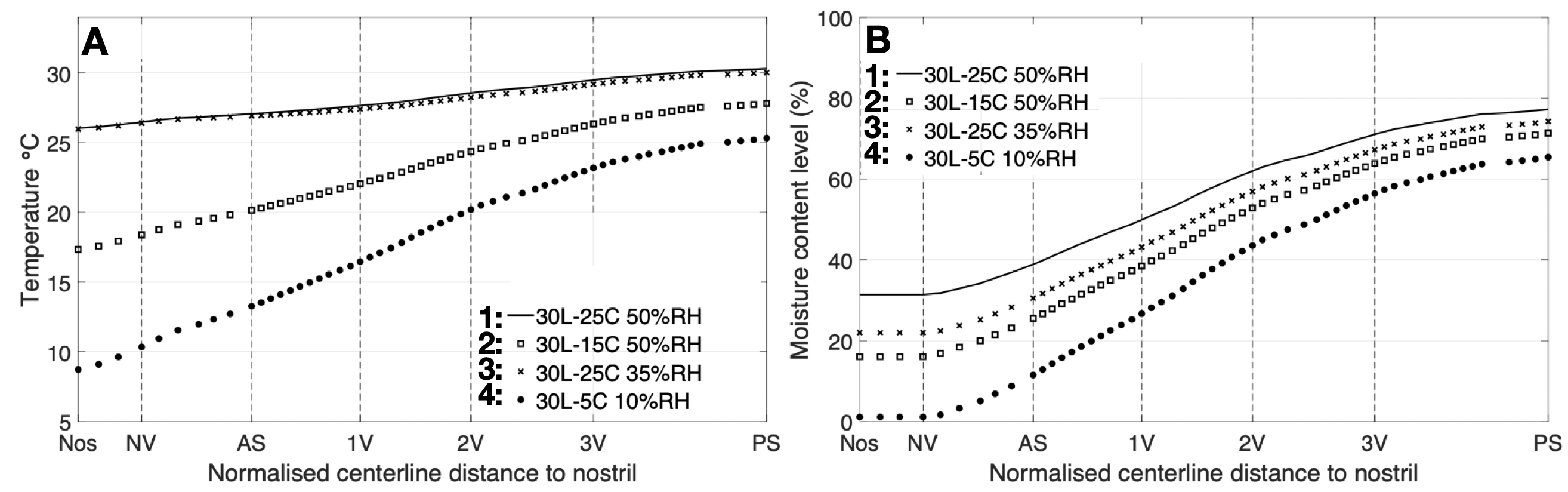


*Figure 4: (A) cross-sectional area averaged temperature distribution inside nasal cavity from nostril to posterior septum of one subject at 4 ambient conditions. (B) shows the same plot but for moisture content level. 100% moisture content indicates condition at 37*$℃$*, 100% relative humidity. The legend shows the 4 ambient conditions described in table 2. From condition 1 to 4, the inhaled air is becoming cold and dry gradually (as indicated by the third column of table 2). All results relate to 30 L/min flowrate only.*

Figure 4A indicates how as the inhaled air becomes cold and dry (from condition 1 to 4), the gradient of temperature increase is higher in the more extreme conditions (condition 3 and 4). It reflects how the high temperature gradient between inhaled air and the wall lining the nasal cavity increases the burden of heating up the inhaled cold air; likewise however it also demonstrates the efficiency of the nasal cavity in warming the inhaled air. Similar behavior also can be found from Figure 4B for the change of moisture content between nostril and posterior septum.

In addition to the above qualitative analysis, we also compared the total amount of added heat and water, calculated by the temperature change and moisture content level change, in the nasal cavity between the region of the nostril and posterior septum as shown in table 3.

| Conditions | 1 | 2 | 3 | 4 |
| --- | --- | --- | --- | --- |
| $\Delta T$ between nostril and posterior septum ($℃$) | 5 | 5 | 10 | 17 |
| Relative change (%) | **0** | **2** | **100** | **240** |
| $\Delta MC$ between nostril and posterior septum (%) | 46 | 53 | 57 | 65 |
| Relative change (%) | **0** | **15** | **24** | **41** |
| Temperature at posterior septum ($℃$) | 30.5 | 30 | 27.5 | 25.5 |
| MC level at posterior septum (%) | 78 | 75 | 72 | 66 |

*Table 3: This table quantifies the relative changes when we simulate more ambient conditions listed in table 2. If we take condition 1 as the reference condition, we can see the large variations between those 4 different external ambient conditions in terms of added amount of heat and water vapor in the nasal cavity between nostril and posterior septum.*

Table 3 shows not only the amount of added heat and water vapor, but also the final temperature at the posterior septum is significantly affected as the external ambient condition becomes more extreme compared to alveolar conditions. If we use condition-one as the reference, it shows both the added heat and water vapor are significantly altered when the ambient condition changes.
